# Editorial for the Special Issue on Micro- and Nano-Fabrication by Metal Assisted Chemical Etching

**DOI:** 10.3390/mi11110988

**Published:** 2020-10-31

**Authors:** Lucia Romano

**Affiliations:** 1 Institute for Biomedical Engineering, University and ETH Zürich, 8092 Zürich, Switzerland; lucia.romano@psi.ch; 2 Paul Scherrer Institut, Forschungsstrasse 111, CH-5232 Villigen, Switzerland; 3 Department of Physics and CNR-IMM- University of Catania, 64 via S. Sofia, 95123 Catania, Italy

Discovered by Li and Bohn in 2000 [1], Metal Assisted Chemical Etching has been finally recognized as an emerging etching technology. In its twentieth anniversary, Micromachines dedicated this special issue to Metal Assisted Chemical Etching. Several acronyms were reported for this process—metal-assisted chemical etching (MACE), metal-assisted etching (MAE), MacEtch—since 2015 the community seemed to agree with the common acronym of “MacEtch”, which was firstly introduced by X. Li [1] to distinguish the unique properties with respect of standard wet-etch and dry-etch techniques. Unlike wet-etch with potassium hydroxide, the MacEtch process is almost independent of crystal orientation and may be used to create a wide variety of patterns, without suffering of micro-loading and scallops of dry-etch. An advantage of the method is the considerable reduction in fabrication costs and complexity with respect to the other techniques. MacEtch is a powerful etching method, capable of fabricating high aspect ratio nano- and micro-structures in few semiconductors substrates (Si, GaAs etc) by using different catalysts (Au, Ag, Pt, Pd etc). Several shapes have been demonstrated with high anisotropy and feature size in the nanoscale: nanoporous film, nanowires, 3D objects, trenches, which are useful components of: photonic devices, microfluidic devices, bio-medical devices, batteries, Vias, microelectromechanical systems (MEMS), X-ray optics etc. With no limitation on large-area and low-cost processing, MacEtch can open up new opportunities for several applications where high precision nano- and micro-fabrication is required. This can make semiconductor manufacturing more accessible to researchers in various fields and accelerates innovation in electronics, bio-medical engineering, energy and photonics.

There are 7 papers published in this Special Issue focusing on several MacEtch applications and fabrication methodologies. Despite the use of metals, which is still the main shortcoming for microelectronics, several manufacturing sectors can benefit of MacEtch. In particular, it seems clear now that the etching capability and the relatively ease of the process are effectively boosting the integration of MacEtch in the microfabrication framework. MacEtch is not only producing porous silicon for new electrochemical applications [2], but also the roughness of GaAs [3] can be tuned for photonic applications. The integration with patterning techniques demonstrated new capabilities from the easy tuning of conical structures [4] to the more complex 3D control by magnetic catalyst [5]. The integration with post-etching processing demonstrated that the etched silicon can functionally serve as template for Al_2_O_3_ nanotubes [6], Pd zone-plates [7] and Au micro-gratings [8]. While fundamentals are still discussed for GaAs [3], silicon requires fine tuning strategies as a function of the application [2,4,5,6]. X-ray optics fabrication seems to be the most advanced application of MacEtch with already in use devices [7,8]. Finally, the challenges and the future perspective of MacEtch as nano and micro-fabrication technology are highlighted in the review article [8]. The future looks bright for MacEtch: the recent increment of publications related to MacEtch indicate a gradual integration of the process in microfabrication protocols, with clear advantages of cost-reduction, nano-features capability and high aspect ratio structures. The only recommendation is to be careful in handling and disposing the hazardous hydrofluoric acid!

I would like to take this opportunity to thank all the authors for submitting their papers to this Special Issue. I would also like to thank all the reviewers for dedicating their time and helping to improve the quality of the submitted papers.
